# Mobile Health (m-Health) in Retrospect: The Known Unknowns

**DOI:** 10.3390/ijerph19073747

**Published:** 2022-03-22

**Authors:** Robert S. H. Istepanian

**Affiliations:** Institute of Global Health Innovation, Imperial College London, London SW7 2AZ, UK; robert_istepanian@yahoo.com

**Keywords:** m-health, mHealth, mobile health, digital health, telemedicine, e-health, telehealth

## Abstract

For nearly two decades, mobile health or (m-Health) was hailed as the most innovative and enabling area for the digital transformation of healthcare globally. However, this profound vision became a fleeting view since the inception and domination of smart phones, and the reorientation of the concept towards the exclusivity of global smart phone application markets and services. The global consumerization of m-Health in numerous disciplines of healthcare, fitness and wellness areas is unprecedented. However, this divergence between ‘mobile health capitalism’ and the ‘science of mobile health’ led to the creation of the ‘m-Health schism’. This schism was sustained by the continued domination of the former on the expense of the latter. This also led to increased global m-Health inequality and divide between the much-perceived health and patient benefits and the markets of m-Health. This divergence was more evident in low and middle income (LMIC) countries compared to the developed world. This powerful yet misguided evolution of the m-Health was driven essentially by complex factors. These are presented in this paper as the ‘known unknowns’ or ‘the obvious but sanctioned facts’ of m-Health. These issues had surreptitiously contributed to this reorientation and the widening schism of m-Health. The collateral damage of this process was the increased shift towards understanding ‘digital health’ as a conjecture term associated with mobile health. However, to date, no clear or scientific views are discussed or analyzed on the actual differences and correlation aspects between digital and mobile health. This particular ‘known unknown’ is presented in detail in order to provide a rapprochement framework of this correlation and valid presentations between the two areas. The framework correlates digital health with the other standard ICT for the healthcare domains of telemedicine, telehealth and e-health. These are also increasingly used in conjunction with digital health, without clear distinctions between these terms and digital health. These critical issues have become timelier and more important to discuss and present, particularly after the world has been caught off guard by the COVID-19 pandemic. The much hyped and the profiteering digital health solutions developed in response of this pandemic provided a modest impact, and the benefits were mostly inadequate in mitigating the massive health, human, and economic impact of this pandemic. This largely commercial reorientation of mobile health was unable not only to predict the severity of the pandemic, but also unable to provide adequate digital tools or effective pre-emptive digital epidemiological shielding and guarding mechanisms against this devastating pandemic. There are many lessons to be learnt from the COVID-19 pandemic from the mobile and digital health perspectives, and lessons must be learnt from the past and to address the critical aspects discussed in this paper for better understanding of mobile health and effective tackling of future global healthcare challenges.

## 1. Introduction

What is mobile health or (m-Health)? For nearly two decades, this important question has been the subject of numerous debates, discussions, speculative analysis and studies. Many of these brought either incomplete, vague or even erroneous answers to this key question. The premise of most of these answers were based on the interpretation and the understanding that mobile health is an adjunct concept associated with the technological and computing traits of smart phones to enable healthcare connectivity and improved delivery services. These powerful traits of the smart phones were utilized effectively and widely via numerous tools and solutions centered on the smart phone health applications (Apps) and their connectivity ecosystems. These largely commercial systems were successfully applied in all shapes and formats to many healthcare, diagnostics, monitoring, wellness, social, and behavioral healthcare domains. Yet, this narrow yet popular interpretation of mobile health is foremost based on the consumerization and the monetization opportunities offered by smart phones in all these areas. However, this m-Health reorientation from the post smart phone era is entirely incompatible with the basic scientific principles of mobile health as were originally envisioned. In order to provide a more succinct and scientific answer to the above question, it is important to understand that the drivers for this reorientation are the ‘known unknowns’ associated with the origin and evolution of mobile health. These were not appropriately or widely understood or disseminated. The reorientation of mobile health towards the singular smart phone centric format was the result of the ignorance of the origin of mobile health, whether deliberate or not, which was initially carried out systematically and meticulously by the leading global corporate telecommunication and IT conglomerates who have shown an early interest in this area, and identified the massive potentials for their markets and businesses first and foremost.

For more than a decade, numerous m-Health systems centered on smart phone applications were applied to a large volume of clinical studies and evidence-based studies conducted globally. In many of these studies (with few exceptions in some applications), the clinical and economic end results proved to be either limited or with no tangible difference. These included, for example, the lack of rigorous evidence on the efficacy, efficiency, cost, global outreach, patient acceptability, privacy, security and many other challenges. These remain largely under the cliché of ‘further research and evidence is required’. Yet, no real alternatives to these systems were presented or proposed. This is because of the powerful and well-established global markets and penetration established by smart phone m-Health applications. From the developing world perspective, and although the proliferation of smart phones and usage of mobile technologies is extensive in these countries, the much hyped benefits of these m-Health systems and their market-driven applications aimed to address the many healthcare challenges in the world’s poorest regions, such as improvement in health inequality, literacy, bridging the care gaps and many other challenges, remain largely modest and unattainable.

The recent COVID-19 pandemic with its devastating public health, human, social and economic impact exposed many limitations of the so-called ‘transformative’ capabilities of the ‘digital health’ applications developed in the wake of this pandemic. Most of these recently retitled m-health applications were developed in response of the pandemic. None of these was thought or implemented prior the pandemic to provide the necessary preemptive digital global shielding or appropriate smart guarding mechanisms against its eventual wider spread with all its human, health and economic consequences. Many of these pandemic era apps and the solutions proposed were permeated around the smart phone market models. Most of these were based on the intrusive contact tracing, symptom detection, early diagnostics, and other pandemic responsive applications. However, with the few exceptions of the mandatory imposed COVID-19 digital contact tracing apps and vaccination passport applications, the majority of these were either unpopular or not widely applicable or practically effective. Furthermore, the many security and privacy issues of these applications still remain a major debatable concern and threat to the data privacy of the users in the midst of this continuing pandemic.

Nevertheless, some of these market-driven m-health applications provided some tangible clinical benefits and evidence-based impact within the clinical and wellness areas, but mostly within selected patient populations and care settings.

In order to understand these complex issues, this paper is structured into the following sections. In the next section, the answer to the key question of ‘what is mobile health or (m-Health)?’ is presented and clarified from both a historical and a literature evidence-based perspectives. It presents the beginnings of mobile health and the core technological and scientific pillars of m-Health. This origin, together with the key milestones associated with its evolution, are also discussed. These also present the key developments leading to the creation of the m-Health schism established after the introduction of the first generation of smart phones in 2007. A critical view and analysis on the contradictions associated with this beginning, and the original definition together with an analysis of the subsequent definitions widely cited since then, are also presented. The validity of these definitions from the historical, taxonomical and ontological perspectives are discussed. These contradictions contributed to the eventual reorientation of mobile health. The third section presents a detailed analysis on the role of WHO in these definitions and the different classifications of mobile health (m-Health). It highlights the relevant shortcomings and contradictions embedded in these definitions and classifications. It questions their validity, and the methodologies used in these classifications of m-Health, and also arguing the questionable assumption of the inclusion of m-Health as part of e-health.

In Section 4, the rapprochement between mobile health and digital health are clarified and presented.

The case for the frequent and interchangeable usage of these two terms is discussed. The unclear distinction and the ambiguous understanding on the differences between the two terms, especially from a smart phone centric perspective, are analyzed. Furthermore, different representations of digital health and its correlation with m-Health and the other standard information communication technologies (ICT) for the healthcare domains are also presented to clarify the issues outlined above.

The issues presented in this analysis warrant radical rethinking of the global understanding and the future outlook on mobile health, and how it can be better understood from a scientific rather than an exclusive market view. These critical views, if continued to be neglected, will lead to further mobile/digital health inequality, missed opportunities of global transformative healthcare benefits, and more market-driven reorientations associated with misguided benefits and limited outcomes to patients and users alike. The next section presents a critical perspective on the key drivers of the mobile health reorientation. It presents the case of the most influential stakeholders that have impacted on this reorientation. Those are likely to impose the same influence on digital health as it evolves, and mostly likely to reshape or reorient the future of this area as well.

The final section discusses the different factors that swayed the scientific progress of mobile health to date, leading to the current and uncertain status quo. The paper concludes with some important suggestions that advocate for the radical rethinking of mobile health based on and guided by the science of m-health, and preferably, but not exclusively, outside the existing smart phone sphere of influence. If this vision is allowed to prevail and is better understood, especially by the global health policymakers and health institutions, scientists, and other interested stakeholders, it can, and only then, radically reverse the status quo of mobile health. These can ultimately create vast opportunities of change towards more affordable, viable and clinically effective m-health systems for all and not for the few. It is only then that the truly transformative traits of m-health can be translated to the more effective and globally impactful outcomes of the current and future global healthcare challenges.

## 2. What Is Mobile Health (m-Health) in a Nutshell?

**Figure 1:**
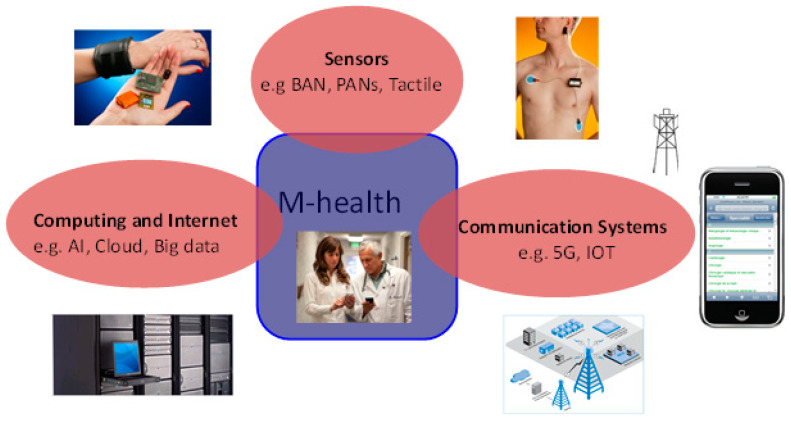
The basic pillars of mobile health (m-Health) (Adapted with permission from Istepanian et al. ([1,3]).

To answer this question, it is best, first, to understand the basic DNA of mobile health (m-Health). This can be scientifically explained by revisiting the imperative notes that set the motion to establish the original concept and its subsequent definition [1,2]. The principal pillars of m-Health are shown in Figure 1.

These pillars were formulated and embedded in the first definition of mobile health: ‘*mobile computing, medical sensor and communications technologies for healthcare*’ [2,3]. This definition remains the acceptable notion and the cornerstone in understanding the concept of mobile health. These pillars underpin the scientific and technological principles of computing, communications and sensing technologies applied for healthcare that encapsulate these basics. The details of these and other imperative notes are described elsewhere [1,2,4].

Some of the ‘m’s widely attributed to m-Health include:

Mobility: The mobility aspects of m-Health are characterized with the utilization of the various modalities of mobility that aimed to improve healthcare access, increased efficiency, and potential cost reductions. However, these were largely applied within the context of m-Health smart phone applications.

Monetary and Markets: Mobile health created unprecedented markets and business ecosystems on a global scale. These largely comprise the digital innovations linked to the numerous smart phone mobile health application systems and services, and utilized for a wide spectrum of healthcare and wellness markets.

Medical evidence: Large-scale medical and clinical evidence of m-Health applications and interventions remain largely scrutinized and under the radar. Although some of these applications proved clinically effective and beneficial, many others still remain debatable with no clear evidence. 

### 2.1. The Evolution of m-Health and the Key Milestones: Progress and Reorientaion

The fundamental principles of m-Health as described above were radically reoriented in the post smart phone era, and mark the current *modus operandi* of mobile health as it has been widely understood since then. The introduction of the smart phone centric mobile health applications and the market ecosystem that followed this reorientation undermined the basic scientific and technological principles presented earlier. These not only refocused the above principles through the prism of smart phone technologies and tools, but also diverted, in a globally successful way, the concept towards market priorities by encapsulating these principles within the smart phone applications (apps) domain or sphere of functions. Consequently, this reorientation process was hyped by the much-promised global transformation, outreach and improved healthcare delivery priorities of these mobile health systems.

**Figure 2:**
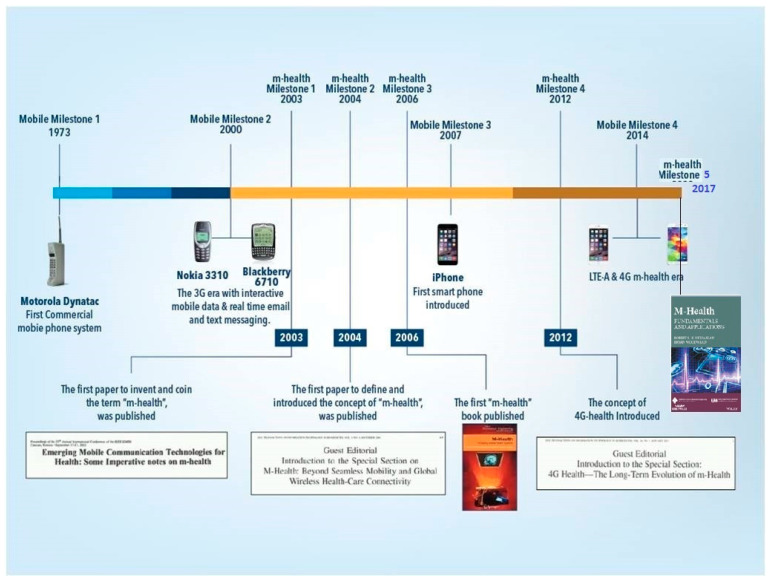
The evolution and key milestones of mobile health (2003–2021). (Adapted with permission from [1] 2017 Hoboken, NJ, USA: John Wiley & Sons).

Figure 2 shows the timeline of the evolution of mobile health since 2003. It highlights the timings of the first definition and the relevant scientific and the technological milestones and developments that impacted its evolution since 2007 [1]. The importance of these milestones in this context are described elsewhere [1].

As shown, the most impactful of these was the introduction of the smart phone (iPhone) in 2007. This technological breakthrough ushered in a new, yet controversial, era for m-Health, and consequently created the ‘*mobile health schism*’ with two distinct, but asymmetrical axes [1]:

(i) The ‘market driven m-Health’ or the smart phone centric models and ecosystems.
(ii) The ‘science of m-Health’ as presented in the original core principles outlined earlier.

Whilst the success of the former is driven by the massive global markets created as a result of the unprecedented proliferation of smart phone m-Health applications (apps), the latter remained less understood or widely discussed [1]. It is because of this schism that mobile health was and remains widely interpreted by the former axis. This interpretation became increasingly tangled by the increasing and controversial clinical debates, supported by numerous studies that questioned whether these m-Health apps can deliver many of the much-hyped transformative and advocated outcomes and promised benefits. The COVID-19 pandemic proved the validity of this view and analysis, especially on the inability of these systems to effectively predict, respond to and mitigate global public health and catastrophic emergency situations. However, there are some valid arguments on the evidence base, clinical and economic benefits provided by some of these m-Health applications, but only in specific healthcare, wellness and patient care settings [1,5,6,7] The global scaling-up associated with robust clinical and economic evidence on a wider scale, and the sustainability of their usage, are yet to been proven [8].

As shown in Figure 2, the second decade has seen continued reorientations towards the further expansion of the m-Health application ecosystems, with less attention paid to the core scientific, clinical, economic, security, privacy and other critical aspects. Over the last decade, hundreds of thousands of these mobile health applications (m-Health apps) have been developed and introduced annually. These range from applications developed by small-to-medium enterprises to globally marketed systems developed by giant high-tech and IT and pharmaceutical conglomerates [7]. The most successful of these applications were largely sustained by the massive injections of capital investments, corporate support, and rigorous marketing strategies. These efforts eventually paid off, creating globally successful m-health powerhouses from leading stakeholders behind these developments. However, strong evidence is yet to be seen on the scaling-up of these applications, especially in the developing world and poorer regions that face many health challenging environments [7,8]. It is well known that the earliest attempts at this reorientation were initiated by global telecommunication providers and corporate philanthropic organizations. These sensed, early on, the potential of these m-Health markets, and the benefits to be gained from these opportunities [9]. These were followed by the profiteering IT and Internet giants, for the profit of healthcare providers, major healthcare insurers, and global pharmaceutical conglomerates. The business opportunities and the massive returns of investments (ROI) offered by the m-Health markets and services were tempting for these corporate organizations.

During the second decade of this evolution process, and as a result of this vast global corporate popularity and thrust, the support of the United Nations (U.N.) and its global institutions, such as the World Health Organization (WHO), International Telecommunications Union (ITU), as well as other global organizations sponsored by the telecom industries, such as (GSMA), followed suit and was eventually secured.

However, many of the earliest global mobile health initiatives and the mobile health strategies embraced by these established global institutions and organizations had limited impact, largely due to the dependency of these initiatives on potential market-driven models and opportunities, rather than real outcomes and tangible benefits. These largely neglected the scientific aspects of the solutions used. Furthermore, most of these remained largely localized, and within the pilot spheres, with no wider prospects to successfully scale up, especially in the poorer regions of the developing world. Numerous studies highlighting these critical issues were published [6,7,8,10]. This reorientation process led to the inevitable creation of the mobile health schism described earlier. Most of these studies have repeatedly addressed the challenges associated with these initiatives, but without pinpointing to the original causes or proposing viable and practically applicable alternatives. There are many other examples and critical aspects that can be highlighted on this matter; however, presenting the details of these is beyond the scope of this paper. Most of the consumerization issues of mobile health, particularly in the areas of wellness, disease monitoring and management, and wearables developed, increased the market asymmetry of this schism at the expense of the science. Whilst this market reorientation process continued unchallenged, many of the studies that seriously questioned these aspects, including larger clinical evidence, patient benefits, long term applicability, efficacy, cost effectiveness, behavioral change, privacy and security challenges, still remain debatable and uncertain [6,7,8].

In the midst of this quagmire, the mobile health that was once hailed ‘as the greatest and the biggest technology breakthrough of our time’ [11], and as stated by World Health Organization as having ‘the potential to transform the face of health service delivery across the globe’ [12], remained largely elusive. Therefore, what has changed since then and why are we witnessing the recent shift from m-Health to digital health? The answer to this question, which has remained undiscussed, is multifaceted and complex. Some of these issues are discussed next, whilst others are related to the original underpinnings of mobile health and the misguided interpretations of the concept that were described earlier.

These controversial ‘known unknowns’ were not discussed or disseminated in open literature *prima facie* until now, and are presented and discussed next.

### 2.2. Mobile Health (m-Health): Definitions, Classifications and Contradictions

In order to clarify some of these ‘known unknowns’, the origin and beginnings of mobile health (m-Health) need to be clearly presented and discussed thoroughly, based on the scientific literature and relevant evidence-based facts.

As shown in Figure 2, mobile health originated in the seminal and pioneering work of Istepanian et al. in 2003. This work has been widely acknowledged by numerous scientific and literary publications since then [1,4,13,14,15,16,17,18]. However, many of the ad hoc follow-up definitions and self-styled derivative interpretations of mobile health that followed this pioneering work fundamentally altered and eventually misguided the original interpretation and scientific underpinnings of the concept.

To illustrate this, Table 1 shows a chronological perspective of the first definition of mobile health (m-Health), and some subsequent and widely cited literature definitions that followed the original definition. It is important to note that this list is not an exhaustive or full review of all mobile health definitions in the literature to date, but is an illustrative exemplar presented in this article for the completeness of the discussion. A complete compilation and etymological analysis of these and other m-Health definitions is beyond the scope of this paper and is subject to future work in this area.

The definition of m-health by the World Health Organization (published as part of their Global Observatory for eHealth (GOe) compendium [12,22,23] is the most critical definition of these and warrants further analysis. The WHO in this compendium defined m-health as a ‘*medical and public health practice supported by mobile devices, such as mobile phones, patient monitoring devices, personal digital assistants (PDAs), and other wireless devices*’ [12,20,21]. First, it is hard to understand the reasons why neither the original work nor the definition of mobile health were not cited or referenced in this publication, considering the voluminous literature that cited the original work and definition in the time period that elapsed between the original definition publication and the publication of this WHO compendium [2,3,4]. This important, yet evident, literature gap and the unjustifiable omission of the original work remains both unexplainable and undiscussed, perhaps even unethical. It represents an example of the ‘known unknowns’ and is subject to future work and analysis in this area.

However, the analysis of this widely accepted definition of mobile health indicates that it narrowed the area to the confines of the mobility, wireless access, and health monitoring domains. It also used some of the now defunct and obsolete terminologies (e.g., personal digital assistance), and most critically, it contributed to the misunderstanding and reorientation of the concept. Furthermore, this definition lacked some important taxonomical and ontological aspects, which we present next:

(i) The taxonomical perspective: For evidence of this issue, we refer to a classification study that was based on a selection of ten mobile health definitions, classifying the taxonomical aspects of m-Health into four taxonomical dimensions [24]:
  - Healthcare practices;
  - Technological modality of the mobile device;
  - Intended user group;
  - The stakeholders.

Each of these dimensions had further sub-categories included in each of these dimensions.

(ii) The ontological perspective: For evidence of this perspective, we cite another ontological mapping study of selected m-health definitions that classified these definitions into three sub-dimensions, each having their own subcategories relevant to each of these dimensions [25].

This ontological mapping was essentially developed to assist the visualization process of the m-Health landscape and to enable the experts in this area to analyze and map the different m-Health systems to the best practice and identify any gaps in these systems. These dimensions were identified as [25]:

- Structure: This dimension describes m-Health in terms of the hardware, software, networks, data, processes, people and policies associated with m-Health systems.
- Function: It describes the acquisition, storage, retrieval, processing, and distribution functions of m-Health systems.
- Semiotics: These include the data generated from the health records and the information and knowledge associated with these data.

These taxonomical and ontological aspects indicate the clear shortcomings associated with the WHO definition. They warrant valid, but strong scientific arguments for the serious revision of this decade-old definition, and for the adoption of a more inclusive and accurate definition based on the original principles and definition of m-Health, as described above. However, the subsequent, but derivative definitions were used by other WHO publications [20,21], as shown in Table 1. The content of these definitions remains largely as recycled versions of the earlier definition. Furthermore, more recently, these and other controversial issues associated with mobile health seemed to have compounded the WHO to shift their policy towards the usage, instead the term ‘digital health’ [20,21]. The underlying factors and the reasons for this policy shift and the ‘shying away’ from the use of mobile health remain unclear and unexplained. Due to the relevance and importance of this issue, an initial analysis with a critical view of this terminology and policy shift is described next. These issue remain largely ‘known unknowns’ and important, yet undiscussed topics that required further analysis and future work.

## 3. The WHO Definitions and Classifications of Mobile Health: A Critical View

In order to clarify the above and other relevant issues, it is important to first examine closely the WHO’s classification of m-Health as part of the e-health domain, as cited in their WHO Global Observatory for eHealth (GOe) compendium [12], and other publications relevant to this compendium [22,23]. The origin and the scientific basis or justification of this classification remains unknown to date. It is also difficult to locate or argue the methodological, chronological and relevant taxonomical approaches, if there are any, that were used or applied to create this classification and assumptions. They are clearly absent and none are presented in these documents. Furthermore, this compendium did not present or cite any relevant scientific or taxonomical studies to justify and clarify the basis of this classification or justify its formulation.

**Figure 3:**
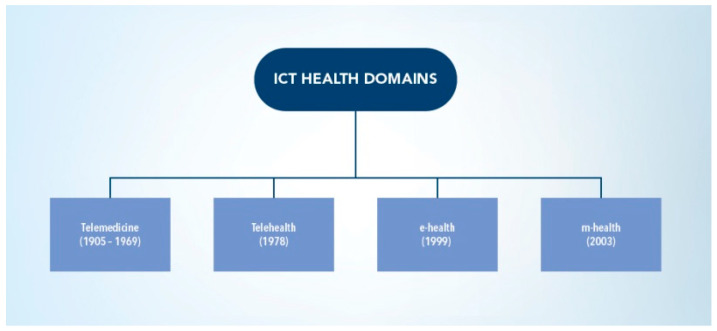
Key domains of ICT for health (Adapted with permission from [1] 2017 Hoboken, NJ, USA: John Wiley & Sons).

However, this classification did contribute further to the reorientation, the ambiguity, and the misunderstanding of the science of m-health. Below are some critical, but valid arguments against this classification. These critical issues warrant further research and investigative work on this misguided and erroneous classification:

(i) This decade-old WHO classification that assumes the inclusion of m-Health as a sub-domain of e-health seems to contradict the WHO’s own subsequent interpretations of m-Health and its correlation with digital health [22,23]. The ambiguity of this interpretation leaves many unanswered questions and is not clear as to the valid scientific aspects of the relevant methodologies and relevant literature approaches that were followed, if any, in this classification process.
(ii) This classification contradicts the well-known and widely cited chronological taxonomy of the standard Information and Communication Technologies (ICT) for the healthcare domains [1]. The ICT taxonomy is shown in Figure 3. In this widely used and cited taxonomy, m-Health is classified as the fourth ICT for the healthcare domain, alongside the other canonical domains, which are telemedicine, telehealth, and e-health [1,4,17]. As shown in Figure 3, these ICT for the healthcare domain are well established and based on their chronological development and relevant technological evolutions, since the introduction and advent of the earliest telemedicine systems in the late 1960s to the subsequent technological and scientific advances that led to the introduction of each of these standard domains. Furthermore, the original premise of this taxonomy is based on the fact that each of these canonical domains are independent and non-inclusive with the others. These have each specific and unique healthcare delivery applications areas and specific technological tools and infrastcture, as introduced in this evolutionary process. This contradicts the above WHO sub-classification of both domains.
(iii) This classification also presents a counter argument from the perspective of e-health and its mobility aspects. It is known, from the various definitions of e-health, that this domain is a combined representative of many Internet-centric platforms developed for different healthcare service delivery mechanisms and their data access tools. These include, for example, electronic health record (EHR) systems, electronic patient records (EPRs), electronic medication portals and other technologies under the e-health umbrella term [1]. However, technological advances—associated with seamless mobility, mobile Internet access, and the developments in the global wireless/cellular connectivity (all fall under the m-Health domain), combined with the emerging Internet-centered data access platforms and associated technologies (cloud, block chain, big data, etc.)—are fundamentally shifting the traditional computing e-health platforms defined two decades ago towards more mobility access and connectivity. These are subsequently becoming contained within the mobility and their computing technological spheres. It thus is a valid argument that most of the traditional e-health systems have migrated to their mobility platforms or towards the (m-health) domain and not the reverse, as stipulated in the classification above. This argument thus strengthens the case for a reverse classification, if we assume the validity of the above definition by WHO, and since most of the current e-health platforms and access technologies are becoming increasingly more used and applicable within the m-Health technology domain.

All the above, as well as many other issues warrant transparent debates and more scientifically open discussions to understand the validity of these classifications better. These classifications need to be based on succinct, scientifically led and literary-based methods that avoid being non-inclusive or biased. These future studies can provide a better understanding of mobile health with more scientific insight by going back to its basics, and not to continue in this recycling path of possibly flawed, inaccurate classifications and outdated definitions.

## 4. The Rapprochement between Mobile Health and Digital Health

In recent years, there has been an increasing trend in the use of the term ‘digital health’ in conjunction with mobile health. This relatively new trend has created vast conjectures on what digital health is and how it relates to mobile health. The rapprochement between the two disciplines remains unclear, and the distinctiveness between the two disciplines remains ambiguous to date. In order to understand this ambiguity from the m-Health perspective, it is important to present the origin of the digital health term first. The beginning of the exact term ‘digital health’ remains largely uncertain. Some literature reviews cite the origin of the term as ‘digital healthcare’, which was used during the Internet boom of the late 1990s [26]. However, other references associate the beginning of ’digital health’ with an early definition of ‘digital healthcare’ as ‘*largely encompassing internet-focused applications and media to improve medical content, commerce, and connectivity*’ [27]. However, ‘digital health’ as it is termed at present, seems to be popularized interchangeably in conjunction with mobile health (m-Health). The ‘known unknowns’ presented next have not been widely discussed or presented in the open literature prima facie until now and can be subject to further research and work.

To date, similar to m-Health, there are numerous institutional and individualized definitions of the term digital health. These include, for example, the WHO definitions [21,28,29,30], the U.S. Food and Drug Administration (FDA) definition [31], the Healthcare Information and Management Systems Society (HIMSS) [32], and many others [33]. A list of these and other widely cited definitions are shown in Table 2.

The task of classifying and reviewing all these definitions is beyond the scope of this work and is subject to further work in this area. From the author’s perspective, digital health can be best interpreted as ‘*the convergence and utilization of digital sciences and technologies for healthcare improvement and the digital transformation of medicine*’. Assuming that the most plausible beginning of digital health can be traced back to the early definition of ‘digital healthcare’ [27], a simple comparison of this definition with the more recent definition by WHO of ‘digital health’ concludes that the early definition is neither a representative nor an accurate interpretation of the current understanding of digital health. The WHO, in their digital intervention guidelines, states that digital health is rooted in e-Health, which uses Information and Communications Technology (ICT) to support of health and health-related fields. However, this statement does not refer to other standard ICT health domains, as discussed earlier. This makes such an interpretation questionable and possibly invalid. However, the WHO also states that digital health involves mobile health, which uses mobile wireless technologies for health, as explained earlier [21].

These diverse interpretations fuel further speculative uncertainties and term ambiguities, especially between mobile health, e-health and digital health.

To illustrate this, we cite the WHO guidelines on digital interventions, in which digital health is defined as ‘encompassing e-health (which includes m-health), as well as emerging areas, such as the use of advanced computing sciences in “big data”, genomics and artificial intelligence’ [21]. This interpretation is in line with the earlier WHO classification of e-health and m-Health, as discussed in the previous section. However, the U.S. Food and Drug Administration interpreted digital health more broadly as ‘the broad scope of digital health includes categories such as mobile health (mHealth), health information technology (IT), wearable devices, telehealth and telemedicine, and personalized medicine’ [31].

More recently, the WHO has published their global strategy on digital health (2020–2025), defining digital health as ‘*The field of knowledge and practice associated with the development and use of digital technologies to improve health*’ [28]. However, in this strategy document, an attached footnote to this definition or interpretation states the following: ‘*Document EB142/20 on mHealth, noted by the Executive Board at its 142nd session (see document EB142/2017/REC/2, summary records of thirteenth meeting, section (2)), stated that “Today the term «digital health» is often used as a broad umbrella term encompassing eHealth as well as developing areas such as the use of advanced computing sciences (in the fields of “big data”, genomics and artificial intelligence, for example)*’ [28]. Furthermore, the details of the content of this footnote were not clarified, especially on what seems to be a ‘reversal of fortunes’, for the still unknown key policy decision on shifting from m-Health to digital health in such a short space of time. This policy shift was demonstrated by the presentation of this recommendation, but with the term replacement for the subsequent approval at the 73rd World Health Assembly (WHA) in 2020. As a result of this puzzling policy shift, the term mobile health (m-Health) was absent from this strategy document and was excluded from its glossary of terms and definitions. This critical issue, on what seems to be a programed and perhaps deliberate procedural issue mandated by the WHO officials running its newly established digital health department, remains unexplained and poses legitimate questions on the above issues that need to be answered. This important issue becomes more critical, considering the decades-long interest and major support of mobile health (m-Health) by the WHO prior to 2020.

In order to present and clarify the ‘known unknown’ issues associated with this undeclared WHO stealth strategy and reorientation policy of the terms digital and mobile health, the beginnings of this yet undiscussed topic should be studied, which dates back to the 71st World Health Assembly in 2018. In this assembly, a provisional ‘m-health’ resolution (*No. A71/20*) published in a WHO document (*No. EB142/20*) on ‘*mHealth*’ seems to have been provisionally agreed on by the committee preparing this document, and to be presented for final approval by the World Health Assembly [20]. This provisional document reflected, until then, the global importance of m-health as detailed in this draft resolution. However, for a ‘known unknown’, this draft resolution was retitled, with minor changes, as the ‘*digital health*’ resolution (*No. WHA71.7*), and all references to mobile health were removed. It was then presented for approval, and subsequently published at the end of the assembly [30]. This ‘known unknown’ is thus appears to be a mandated by internal WHO procedure, and unspecified policy driven change intended for deliberately retitling the document behind ‘closed-door expert committee’ meetings. The details of these issues remain undisclosed and unjustified to date.

Following this ‘digital health’ declaration, similar procedural changes seem to have been followed, with identical terminology shifts and usage that were subsequently adopted by most of the other global organizations interested in this area. These include the International Telecommunication Union (ITU), International Organization for Standardization (ISO), and many other industry-led global organizations, who were interested in the mobile health area and had used the term for more than a decade prior to the shift.

The timing, the underlying factors, and the real motives of these policy shifts of interchanging terms leave many unanswered, but legitimate, questions on the transparency of these internal policies, and on whether any scientific basis were followed in these internal discussions and committee meetings that would have justified these major global decision-making processes on this matter.

These undiscussed issues perhaps reflect the strict hierarchical and bureaucratic procedures that are followed in the WHO and the other global organizations.

It is possible that opinionated decisions and perhaps undeclared whims or politically influenced factors are associated with these decision-making processes. This suggestion is advanced in the absence of any agreed knowledge, global consensus or understanding of what digital health is and how it differs from mobile health. The latest WHO definitions on digital health need further clarification on the disparity of these definitions, and also how they differentiate digital health from the well-established domain of mobile health and the other Information and Communication Technologies (ICT) for healthcare domains (telemedicine, telehealth and e-health). The wider understanding of these important issues still remains as a ‘known unknown’.

Some of these classification and terminology issues were addressed in a recent WHO (Regional Office for Europe) policy brief entitled ‘Use of digital health tools in Europe before, during and after COVID-19’ [34]. This policy brief was published in response to the vast health, social and economic impact of COVID-19 in Europe. These and other documents were also published in response to the global criticism on the WHO’s delay in declaring COVID-19 pandemic and on the seriousness of this disease. In addition, there was an absence of any appropriate and effective digital health tools or any preemptive digital epidemiology systems that could provide an effective digitally led shield for an early global warning system, which could had provided important and early alerting and mitigation information tools against the global spread of the SARS-CoV-2 virus. Furthermore, in this brief, the classification of digital health in relation to e-health and m-Health, big data and other areas within the (ICT) standards was presented with a more informed and succinct approach. Unlike the other relevant WHO documents, this classification approach and the examples given emphasize the distinction of these standard domains. It also encompasses the separation principle of the m-Health and e-health domains within the digital health umbrella term. However, this classification contradicts the earlier WHO classifications of m-Health as part of e-health, as discussed earlier [12,22].

To emphasize these critical issues, it is relevant to cite the example from the United States and the FDA’s definition of digital health. It represents digital health within the broader scope that was described above to include the categories of mobile health, health information technology, wearable devices, telehealth and telemedicine, and personalized medicine [26]. This representation of digital health comes closer to the above representation, and leans further to the framework presented next in this paper.

However, these different interpretations, definitions and classifications, including those given by the WHO, provide more ambiguity rather than clear cut clarifications on these important issues, and necessitate further work in these areas. The continued cycle of alternative definitions, interchanging terms, and their respective interpretations perhaps reflects the trend of opinionated, policy-biased, possibly non-scientific and irrational decision-making process tasked with the preparation of these important issues.

The need for a more scientifically succinct and rigorously scrutinized approach to study and formulate these definitions and their interpretations is vital to achieve a globally accepted consensus. These will provide a better understanding of the detailed scientific and technological aspects of these terms, and importantly, their distinctiveness and the domains of their healthcare applications.

### Digital Health Representations and Correlations with m-Health

There is a clear lack of rigorous studies that address the best representation of digital health, as well as a lack of appropriate taxonomical and correlative framework with m-Health and other standard ICT for the healthcare domains. In this section, we aim to address this issue and propose a valid framework that encapsulates these issues.

**Figure 4:**
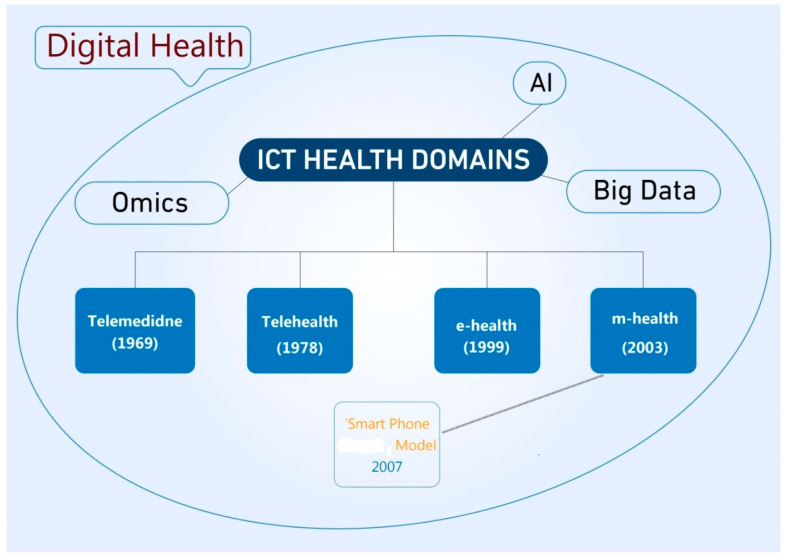
A digital health representation in conjunction with the canonical ICT for the healthcare domains (adapted from Istepanian and Al Anzi (2019) [6]).

If the assumption that the digital health definitions of the WHO, as shown in Table 2, are the most likely and acceptable formats, then two valid interpretations of digital health can be concluded:

(i) The first framework is illustrated in Figure 4 [6]. In this representation, digital health is interpreted as an enclave that encompasses all the canonical and standard (ICT) for healthcare domains of telemedicine, telehealth, e-health and m-Health. This representation matches well with the latest WHO and the FDA interpretations of digital health, as shown in Table 2. This framework can be validated on the basis that it embraces the emerging scientific and technological developments encapsulated within the digital space. These include the many areas that are widely embraced within the digital health sphere, such as Artificial Intelligence (AI), big data science, Internet-Of-Things (IOT), fifth generation (5G) and sixth generation (6G) mobile communications systems, genomics and personalized medicine. Most importantly, this framework maintains the distinctive traits of the individual constituent ICT for the healthcare domains. It also presents the most likely and applicable rapprochement model between digital health and mobile health (m-health) as a separate domain, but as part of the digital health enclave and its constituent domains. Further details of this framework representation are described elsewhere [6].
(ii) A second digital health representation is based on the smart phone centric models identified within the mobile health domain. This representation can be interpreted in Formula (1), shown below:
  Digital Health = m-health (smart phone centric models) + *f* (AI, Big Data, IOT, 5G, Genomics) (1)

This representation interprets the popular understanding of digital health as the smart phone centric representations of mobile health supported by the emerging areas shown. 

However, Formula (1) can also be interpreted as a valid representation of the next generation of mobile health systems or (m-Health 2.0) and defined as ‘*the convergence of m-Health with emerging developments in smart sensors, 5G communication systems with the functional capabilities of Web 2.0, cloud computing, and social networking technologies, toward personalized patient-centered healthcare delivery services*’ [1,6]. This definition can be represented in Formula (2), shown below:

m-Health 2.0 = m-health (smart phone centric models) + *f* (AI, Big Data, IOT, 5G, Genomics) (2)

This m-Health formula is, fundamentally, the evolution of the earlier ‘telecom formula’ presented in Formula (3) [1]:

m-health = mobile (smart phone app) + healthcare delivery service(3)

The above interpretations can be considered by some as argumentative and subject for further analysis and debate. However, they also pose many questions that remain unanswered concerning the fundamentals of digital health, and the missing links between the ‘science of m-Health’ and the ‘science of digital health’ and what the difference between the two is.

Further studies are required to provide the answers to these important, yet still undiscussed issues.

## 5. The Key Drivers of the Mobile Health Reorientation: A Critical Perspective

In this section, we present a critical perspective of the key drivers that are involved in the reorientation process as discussed in earlier sections. This critical analysis is based on the classification of the most effective mobile health stakeholders that have been identified in numerous studies relevant to this area [1].

These stakeholders are likely to impose the same influence on digital health as it evolves and are mostly likely to reshape or reorient the future of this area as well. The most influential stakeholders and drivers that have impacted the mobile health reorientation are:

(i) Mobile health global businesses and corporate conglomerates.
(ii) Mobile health ‘gate keepers’.
(iii) Mobile health consumers.
(iv) Mobile health policymakers and regulators.

### 5.1. Mobile Health Global Businesses and Corporate Conglomerates

The prime mover of these stakeholders is encompassed by global mobile health corporations and their conglomerates. They were largely responsible for leading the global mobile health business and market thrust in the past decade. These powerful conglomerates include most of the global tech, IT and telecommunications providers, smart phone companies, medical devices manufacturers, pharmaceutical industries, m-Health apps and wearable industries and others. Most, if not all, embrace mobile health as a vital sector within their global businesses and fiscal strategies. Their target was and still is to dominate the mobile health consumer and global user base through many of their lucrative healthcare and wellness markets and services.

The products and the services offered by these conglomerates are being increasingly relabeled under the ‘digital health’ umbrella term. These are most likely to refocus on the same provision, but with advanced mobile health technologies and patient centric approaches. The basic modus operandi of these new models is to upgrade or modify their existing m-Health technologies and to remarket them as digital health solutions and services using powerful remarketing strategies such as corporate sponsored studies, meetings and conferences, and indoctrinating social media outlets. These new systems will be largely utilized by the recent advances in areas such as Artificial Intelligence (AI) and Machine Learning (AI/ML), 5th (5G) and the future 6th (6G) mobile and wireless communication systems, the Internet-of-Things (IOT), sensor connectivity, cloud and big data, block chain technologies. Most, if not all, of these marketing tools will be based on the same focal point and based on the next generation of smart phones as the main conduit for these tools and services. These developments will ultimately aimed to scale up the vertical consumer markets and entice the new millennial generation of users to embrace these services. However, the critical clinical evidence base, cost effectiveness, affordability and many other challenges described earlier will again be subject to critical scrutiny and perhaps subject to more rigorous policy and regulatory constraints. These issues will remain open for debate in the foreseeable future, and especially regarding the many emerging global healthcare challenges that need to be tackled effectively.

There is a plausible likelihood that the same clinical, economic, cost and many other uncertainties that plagued the market driven mobile health products will be recycled with retitled digital health products [7,35,36]. These uncertainties include the efficiency, efficacy, frugality, cost benefit, patient and long-term acceptability, interoperability, standards, cyber security, ethical and privacy challenges of these reinvented mobile health systems. The validity of this prediction lies in the overarching principles that are distinctly embedded within the DNA of smart phone centric models and their ecosystems that re being remarketed as digital health systems, and it is yet to be seen if and when any of these tools are able to mitigate and tackle the global healthcare challenges and the barriers discussed earlier.

In particular, the cost and affordability of these market-driven smart tools and products will likely to be the most challenging, especially in the poorer regions and in most LMC countries. These settings and their patient populations do not need ‘fit-for-all’ products tailored and services, but more affordable and frugal m-Health solutions and practical alternatives for their resource-limited healthcare settings. The COVID-19 pandemic has both provided evidence and shown the logic behind these arguments. The failure to demonstrate the impact or effectiveness of these digital health tools, particularly in the poorer regions and low-income settings, was evident during this pandemic. Many of these digital mitigation, tracking and surveillance tools, used in both the developed and developing countries, were either widely inapplicable or unable to effectively lower the level of COVID-19 infections, neither to mitigate the mortality rates, hospitalizations, and lockdowns, as was the case of the costly development of the NHS digital Test and Trace App in the U.K. had shown [37,38]. These tools were also largely not able to effectively narrow the digital divide during the pandemic, or to bridge the health inequalities and negative social determinants among the COVID patients with comorbidities. Although the level of investment in digital health increased substantially during the pandemic, the digital divide increased among the population, including the examples cited from different parts of the developed world [34].

### 5.2. Mobile Health ‘Gate Keepers’

The ‘gate keepers’ in this context are the constituents of influential global institutions, experts, clinicians, scientists, philanthropists, policymakers, social media influencers, for-profit healthcare providers, corporate and business leaders, who subliminally influenced the reorientation process of mobile health. Most were, at some stage, strong advocates of mobile health and its transformative traits and benefits, but shifted their allegiance and flowed with the global tide and change in this new directions, presumably to sustain the massive markets and the profit-making process, but with new retitling.

To illustrate this with an example, we cite the many ‘for-profit healthcare providers’ (FPHCPs), who marketed extensively at some stage the benefits of their mobile health services, based largely user on patient payment plans for the care and product services provided. More recently, the same FPHCPs marketed and recycled more or less the same products and services under the ‘digital health’ banner. The affordability, monetization and effectiveness of these reinvented tools and services will most likely be scrutinized and criticized by the patient advocacy and other groups that oppose the excessive digital health profiteering tactics and profit margins gained by these providers and their associated gate keepers. This will become more apparent, especially with the challenging global economic outlook and the realities created by the COVID-19 pandemic. This pandemic has led to large-scale investments in digital health technologies and established new and unprecedented increase in digital health markets and business opportunities in response to the pandemic, including, for example, digital contact tracing apps, digital surveillance, telehealth, remote patient monitoring, disease diagnostics, and many other applications [39].

Yet, regardless of these vast levels of investment, many of these digital tools and services were either largely unable or ineffective in their response to the severe healthcare challenges posed by the spread of the SARS-CoV-2 virus and its many variants globally. The security and privacy aspects of these digital tools, in particular, faced serious concerns. These were driven by the prospect of the ‘mobile (digital) health surveillance’ age. These and other critical issues remain largely debatable from many clinical, economic and social perspectives. In the midst of the global healthcare challenges and the severe realities created by the COVID-19 pandemic, the role of the powerful ‘gate keepers’ will most likely continue to be surreptitiously influential, aligning these perceptions to serve their powerful beneficiaries and sponsors. This can be interpreted by the same failure associated with their earlier and much hyped rhetoric of their mobile health reorientation strategies, the disproportionate schism and the expectations created by this reorientation. For more than a decade, the same old issues relevant to m-Health have been debated and discussed repeatedly by the ‘key informants’ of the area. These include policy, regulatory, health systems, research, wireless access and networking, funding, m-Health practices and other issues [40,41]. Yet, these and other studies were largely undermined by the absence of the core understanding of the science of m-Health and the causes of this reorientations as discussed earlier.

The harsh human and cost realities of COVID-19 pandemic have driven most of the mobile health gate keepers to seek new alternatives and possibilities, including the usage of the term ‘digital health’. These face-saving alternative strategies are meticulous planned and organized globally to allow the continuation of the same recycled hype and promises, but under a new umbrella term. This misguided process, if allowed to continue, will ultimately fuel the same arguments and critique of the same clinical evidence, global scaling up, cost effectiveness, patient outcomes, and other uncertainties as was the case with m-Health.

There are serious lessons to be learnt from these critical, yet realistic views. The peril in the continuation of and reliance on the same misguided and indoctrinating digital health strategies will ultimately make itself felt in the same flawed expectations and questionable arguments as before. These, if allowed to continue with the same old rhetoric of the powerful traits of the smart phone digital health tools and their transformative benefits, these will ultimately lead to the same questionable and unfortunate cycle of lost opportunities of tangible and impactful healthcare benefits, especially for those most vulnerable and underprivileged populations.

### 5.3. Mobile Health Consumers

This represents the future generations of digital patients and consumer populations. The millennial and digital savvy population will likely to be more informed on their healthcare conditions compared to their parent generations. This is due to the influence of social media, Internet penetration and the rigorous marketing of smart phone applications for health, combined with the excessive consumerization of commercial digital health products and paid for services, especially in the developed world. This generation is likely to be the target of the global conglomerates and the gate keepers, who will aim to rigorously market and popularize these digital health products, gadgets, and gizmos in unprecedented levels. These will mostly manifest themselves in the next generation of smart phone tools and advanced services embedded with these digital health applications and gadgets. As the gap between the rich and the poor increases, so do the digital divide and the health inequality gaps. It remains uncertain how these market and for-profit digital health tools and systems will be able to successfully bridge this gap, and to best crystalize these solutions for sustainable and associated with long term impact and effectiveness globally. It is also important to note that the existing health inequality and increased social and economic burdens in low-and middle-income countries (LMIC), especially in the post COVID-19 world, is likely to increase the current mobile (digital) health disparity, considering the current economic climate. Additionally, the critical health conditions in these LMIC fall outside the existing digital health markets and consumer bubble of the developed world. The much-needed change of the ‘field of dreams’ trajectory will likely face an influential opposition from the other powerful drivers, as discussed earlier.

### 5.4. Governments, Regulators and Mobile Health Policy Makers

The predominant and typical objectives of the governmental institutions and non-profit organizations from the mobile (digital) health perspective are to facilitate the evolving regulatory and policy landscapes and to achieve the successful implementation of different healthcare services using appropriate digital tools and technologies.

In recent years, there have been many global efforts and attempts in these directions. For example, many regulatory and policy recommendations aim to guide the most appropriate digital health interventions and recommendations associated with existing healthcare governance and policy practices. These recommendations, policies and evidence were produced from different global and governmental institutions. These include the WHO [21,28,29], the European Union (E.U.) [42], the U.K. [43] and other countries [44]. Most of these digital intervention strategies and recommendations have the commonality of (smart phone centric) ecosystem denominator for the core technological aspects for the perceived healthcare benefits and objectives. However, the implementation of these recommendations and interventions, and in order to ensure their impactful outcomes, remains to be seen. Although there has been a plethora of mobile health (smart phone centric) strategies proposed in recent years, data on large-scale and successful translation of these interventions in low and middle-income countries (LMIC) remain sporadic and largely unknown. Most of what has been implemented in these countries remains limited and on pilot levels, without proper evidence of large scaling up processes, unclear or questionable economic and cost benefits, and unambiguous patient outcomes [1,10,45].

These issues need to be addressed carefully by the mobile/digital health policymakers, regulators and experts in line with the critical issues addressed earlier. These are also to be developed on truly independent approach, outside the influence of the global conglomerates and their gate keepers as discussed earlier.

## 6. Discussion

The progress in mobile health (m-Health) over the last decade has been unprecedented. This popularization was largely driven by the global markets of m-Health products, supported by the fast-evolving smart phone applications and the prevalence of their relevant digital technologies, but much less on the science and global evidence of mobile health.

This asymmetrical duality originated from the reorientation of the basic scientific and technological principles envisaged at its inception, towards the systematic and powerful subjugations of mobile health through the smart phone applications (Apps) prism. The global pervasiveness and popularity of this version of m-heath and the dominating thought culture behind it, was aided by vast and dominating markets of the m-Heath applications popularized since the introduction of the first generation of the smart phones.

Nevertheless, this global and popular understanding of mobile health has been continuously scrutinized and questioned from clinical, scientific, societal, privacy, economic, among other areas. Numerous clinical studies and pilots conducted within the last decade questioned these smart phone m-Health applications from different perspectives, such as clinical efficacy, cost effectiveness, patient acceptability and usage, larger evidence base, global healthcare benefits, and security and privacy among others.

This historic, yet powerful reorientation of mobile health transported the area to a continuous ‘flux mode’. It made this innovative area increasingly uncertain, scientifically and clinically limited on its impact, but vastly successful commercially. The origin of this conundrum was instigated by the powerful global health corporate conglomerates and their m-health ‘gate keepers’, who established and advocated this reorientation and sold effectively the global of m-Health business and not equally the science behind it. These were driven largely by market priorities and objectives, and less by healthcare benefits and evidence-based outcomes. Whether m-Health is one of the greatest technological breakthroughs of our time, or just another much-hyped healthcare technology bubble that could burst soon, is yet to be seen.

The fundamental causes and drivers for this m-Health schism are the ‘known unknowns’ or the ‘the obvious but sanctioned facts’ presented and discussed in this paper. The crux of these ‘known unknowns’ is the global reorientation of the m-Health landscape through the singular prism of smart phones and their ‘apps’ ecosystems. These have driven the powerful market economies of m-Health for nearly two decades. It is likely that these global trends will continue for the foreseeable future, supported by the continued growth of the global m-Health markets and their profiteering schemes, especially in the wake of the COVID-19 pandemic. These will be supported by the recent developments and the potential increase in the pervasiveness of the smart phones and its connected mobile devices, especially in the rapidly expanding markets of health monitoring and wellness applications.

There have been some modest attempts in the past to highlight these gaps and identify the science of m-health [46]. However, these *volte face* attempts remained imperfect or had limited tangible outcomes to tilt this imbalance. Such modest revival remains ineffective in changing the overarching trends of mobile health, and of what is already a well-established and powerful global market. Furthermore, these research and scientific attempts were based on mostly invalid assumptions on the principles of mobile health. These were studied from the same limited prism and narrow scientific pathways of smart phone centric approaches, and perhaps if not likely driven by specific views, research agendas and funding priorities.

However, some of the benefits of the ‘m-Health app’ centric applications have been reported from different applications and perspectives. As an example for completeness, some of these from the patient and clinical perspective are presented [47]:

- To support clinical diagnosis and/or decision making;
- To improve clinical outcomes from established treatment pathways through behavior change and enhancement of patient adherence and compliance with treatment;
- To act as standalone digital therapeutics;
- To deliver primarily disease-related education.

Other benefits were also highlighted from the ‘digital intervention’ perspective and their strategies for different health and communications disciplines [28,29]. However, in spite of these benefits, the global applicability and scaling of m-Health remains largely limited and debatable. Most of the market-driven m-Health systems are confined to specific healthcare settings within the developed world, with the actual evidence provided so far supported mostly the use of these m-Health interventions in limited, but growing number of clinical scenarios [47].

The recent revamping of mobile health applications as ‘digital health’ will ultimately result in the same cycle of either ineffective or limited evidence-based outcomes. This was widely seen during the COVID-19 pandemic, with the largely imperfect, limited usage and varying outcomes of these applications and tools, developed specifically in response to the pandemic. These were largely based on smart phone application solutions, and mostly financed by private and public investments. There is an urgent need to learn from these lessons and for radical revisions and an overhaul of the current thinking behind these strategies. The need for new and preemptive m-Heath systems that will have much more effective and tangible outcomes in response to this pandemic and other future global health threats and challenges is vital.

Many of the ‘known unknowns’ discussed in this paper that expose the totality and exceptionalism of this orientation of m-health are not widely studied or investigated. These and other traits were not widely publicized or disseminated within the relevant m-health literature. Some of these are presented and discussed next for completeness.

### 6.1. The Age of m-Health Surveillance and Cybersecurity

The current m-Health systems are potentially establishing ‘global m-Health surveillance and cyber security age’. The pervasiveness and global penetration of the m-Health apps, wearables, and other connected mobile devices are implicitly opening a Pandora’s box and leading to the creation of this worrying, if not dangerous, health surveillance age.

These devices and apps are rigorously marketed and sold in their billions by the giant IT, smart phone and other m-Health (digital health) conglomerates to support primarily their global business markets and landscape [39]. However, little is known on how the global usage of the acquired health and wellness data from these devices are facilitating a potential beach of privacy with surveillance and cyber security threats posed to the persons using these devices. The unauthorized hacking, intelligent breaches and the monetization of the health data acquired from these devices is increasing globally. Some of the recent hacking occurrences and vast fines incurred on data privacy and security breaches are examples of such a worrying trend [48,49]. These issues are just the tip of the iceberg and are growing concern. These critical issues from the m-Health or digital health perspective have not been widely discussed, with no large-scale research having been conducted on this topic from an m-Health perspective. There are evident fears from the potential adverse outcomes of such research on the economies sustaining these systems, with possible user backlash and curtailing of the profits gained from these systems [39].

### 6.2. The Human and Behavioral Effects of the Long-Term Usage of Smart Phone Apps

This is another ‘known unknown’ of the current m-Health systems that has not been widely discussed before. Recent work has shown that the usage of smart phones has adverse, long-term behavioral and psychological effects, especially among younger populations [50]. For example, it is well known that the newer 5G smart phones and portable devices have higher harmful radiation effects and emissions levels, but the long-term exposure of these devices on human health has not been widely and rigorously studied yet [51]. The same can be said of the adverse effects of these devices used in digital health wellness and monitoring tools, and their potential adverse effects on long term basis. The negative attributes of social networking, and the damaging psychological effects on younger children and adolescents, are being increasingly advocated with alarming levels worldwide [52,53]. The funding for further clinical research on these and other harmful impacts and the long-term exposure and usage of smart phones and their tools is both timely and important. The outcomes of these studies can lead to new findings that might advocate for healthier and less harmful alternatives to these popular systems.

### 6.3. Ethics of Mobile Health (Digital Health)

The ethics of mobile health (digital health) is another important area that has not been widely discussed and presented. This issues also relates closely to the privacy and security concerns as described above. Some relevant ethical issues such as the minimization of the ethical m-Health risks of the expanding m-Health solutions, the increasing role of anatomy and virtual independency of these systems from standard medical evidence assessment, improved health literacy issues, the individualized empowerment from the participatory m-health solutions and others. However, the strategic overhaul of these and other m-Health ethical aspects is likely to face surmounting challenges and diversified opinions from the policy, economic, governmental, patient, and healthcare provider perspectives. 

### 6.4. The Rapid Developments of Science and Technology

These challenges are representative of the fast-moving developments in computing, sensing and communication technologies. These represent the original pillars of mobile health [1,2,3,4], representing the principal areas that have guided the development in this area over the last decade. However, since these were largely encapsulated within smart phone technologies, they were never fully capitalized outside this technological landscape as discussed earlier. However, in the wake of the COVID-19 pandemic, this reorientation illustrated more fragility than robustness in tackling the impact of this pandemic, and is likely to produce the same outcomes in response to future global healthcare challenges.

There is likelihood that, if the same process continues in the foreseeable future, especially in the age of rapid digital developments or the digital health era, it will most likely sustain the same cycle of globally unattainable, unaffordable and ineffective healthcare benefits, especially in the low-income and underserved healthcare settings. 

Furthermore, these aspects contradict with the main goals set up by the WHO in their latest global digital health strategy and vision, which states that it aims to encourage digital health adoption worldwide with more inclusive and universal care for all, not only the few who can afford it [28].

### 6.5. Understanding the Science of Mobile Health

Understanding the science of mobile health is vital in this overhaul philosophy, and remains the major challenge in any radical change from the status quo. For nearly two decades, mobile health has been debated as whether it represents an important area for the provision of improved and effective healthcare services via the combined science of the three fundamental pillar discussed earlier, or merely translated as medical and public health practice using mobile phones, patient monitoring devices, personal digital assistants (PDAs), and other wireless devices. [22]. Furthermore, this reoriented mobile health and interpretation is still being labeled as an emerging field without in depth understanding of the above important issues [5]. The proponents of these narrow and outdated interpretations of m-Health, representing one side of the schism discussed earlier, are either ignorant or unaware of the scientific principle of mobile health. These important, yet rarely discussed topics need wider and more transparent debates from the scientific and clinical community interested in the future of this area. These debates need also to establish an interdisciplinary framework of the scientific aspects of mobile health rather than the status quo. There are also need for more in depth analysis on how best the science of mobile health can tackle the many global health challenges and future crises from the core scientific aspects and not these narrow understandings and limited scopes.

### 6.6. Curtailing the Effect of the Mobile Health Gate Keepers

It is likely that the next generation of ‘gate keepers’ will aim to sustain the *modus operandi* in the digital health era, for reasons that are evident, which mostly pertain personalized benefits and corporate sponsorships. In particular, these strategies will focus on the continuation and enhancing the exclusivity of the recycled rhetoric of the smart phone apps ecosystems, supported with embedded intelligent/AI tools for enhanced efficiency and efficacy of these systems. These will be widely marketed and advocated in next few years and used widely to redefine the new era of digital health. The corporate mobile health gate keepers will reconfigure their earlier business and market strategies and remold these within new digital health sphere of services and products. The radical overhaul and curtailing the impact of the gate keeper and their influence is vital for the future of m-Health and its true global impact and transformative benefits. New policies and rethinking approaches are much needed to provide more inclusive and affordable mobile health or indeed new digital health solutions and systems that transcend the current *modus operandi*.

These can be thought of as more globally pervasive, clinically effective and widely applicable, and affordable with truly outreach capabilities. These newly developed m-Health systems also need to be sustainable in the long term, with greater healthcare benefits, rapidly deployable, and particularly adaptable and preemptive to the future global health challenges and threats.

## 7. Conclusions

The paper presented some of the ‘known unknowns’ and undiscussable issues of mobile health (m-Health). These include presentations on the beginnings, original definition, and the fundamental pillars of mobile health.

The paper also presented the global reorientation of mobile health following the inception of the smart phone technologies. This reorientation has led to the widely popular understanding of mobile health, viewed through the exclusive prism of the smart phone tools and applications or (m-Health Apps) singularity. The evolution milestone by this powerful technological breakthrough represented a fundamental diversion in the evolution of this area, acting as a blessing and a curse. The blessing was in the popularization of m-Health to unprecedented levels, with vast global markets that encapsulate the pervasiveness and applicability of the smart phones for numerous applications and clinical areas. The curse was that it placed mobile health into a continuous flux of uncertainties, by creating the mobile health schism between its science from one end and markets from the other. This divergence narrowed the outlook of this important area to global ‘m-Health Pilotitis’ landscapes, with lesser prospects of its much-hyped benefits, scaling up and impact on the global health scene.

This stealth reorientation has been continuous for more than a decade and will continue for the foreseeable future. This version of mobile health will further inhibit its true healthcare benefits and global transformative aspects. This trend can only be curtailed with the establishment of a more scientifically based m-Health-biased foresight and a new overhaul against this reorientation. The re-introduction of digital health as a conjecture to mobile health is perhaps a reflection and outcome of this reorientation.

Many of the critical ‘known unknowns’ and undiscussed issues associated with this reorientation are also presented and discussed. The basic philosophy presented in this paper is based on looking for the scientific alternatives outside the smart phone box, and beyond the m-Health Apps sphere, and more into the scientific aspects translated by the science behind the three pillars of the concept. This new thought needs to be widely discussed and examined further by experts, clinicians and scientists and other ‘informants’ interested in this area. This process must be reinvigorated by establishing a global alliance among scientists, research institutions, global experts and clinicians to re-establish the area on these scientific basis and understanding, and not to leave it exclusively to be driven by the global corporate influencers and mobile health (now digital health) gate keepers, who surreptitiously contributed to this schism and indoctrinated this reorientation into the global fabric of the area. The ‘known unknowns’ presented in this paper exposed some of these undiscussable issues and translated these with some initial thoughts for further work and research in this area. The new overhaul of mobile health and the scientific based outlook will be capable of balancing the fragile status of the increasing healthcare costs, quality, access and the increasing global health challenges facing humanity. The lessons learned from the COVID-19 pandemic from a mobile health (or digital health) perspective and the modest or imperfect ‘global digital’ responses of the tools used during this pandemic need to be scientifically reviewed, studied and fundamentally revised in order for such an alternative vision of mobile health to be more effectively established and implemented. However, this new science-based vision should not be a pretext for the recycling of the same reorientation approaches used over a decade, including a term change, but on novel scientific and technological frameworks that can provide a truly global mobile health access, affordable, and real quality care in the midst of increasing global healthcare challenges.

## Figures and Tables

**Table 1:** A chronological perspective of the most cited mobile health definitions (2003–2020).

Definitions of Mobile Health (m-Health)—A Chronological Perspective	Source
Mobile computing, medical sensor and communications technologies for healthcare.	Istepanian et al. (2003, 2004) [3,4]
Using mobile communications—such as PDAs and mobile phones—for health services and information.	Vital Wave Consulting–United Nations/Vodafone Foundation (2009) [9]
A subset of eHealth, using mobile devices to deliver health services to the patients.	Michael (2009) [13]
The delivery of healthcare services via mobile communication devices.	m-Health Summit Foundation for National Institute of Health (2009) [19]
Medical and public health practice supported by mobile devices, such as mobile phones, patient monitoring devices, personal digital assistants (PDAs), and other wireless devices.	World Health Organization (2011) [12]
The use of mobile computing and communication technologies in health care and public health is a rapidly expanding area within e-health.	Free et al. (2013) [5]
The use of mobile wireless technologies for public health.	World Health Organization (2018) [20]
The use of mobile and wireless technologies to support health objectives.	World Health Organization (2019) [21]

**Table 2:** Examples of digital healthcare/digital health definitions (2000–2021).

Definitions of Digital Healthcare/Digital Health—A Chronological Perspective	Source
*Digital healthcare: largely encompassing Internet-focused applications and media to improve medical content, commerce, and connectivity*	Frank (2000) [27]
*Digital health* *encompassing e-health (which includes m-Health), as well as emerging areas, such as the use of advanced computing sciences in ‘big data’, genomics and Artificial Intelligence*	World Health Organization (2019) [21]
*The broad scope of digital health includes categories such as mobile health (m-Health), health information technology (IT), wearable devices, telehealth and telemedicine, and personalized medicine*	U.S. Food and Drug Administration [31]
*Digital health connects and empowers people and populations to manage health and wellness, augmented by accessible and supportive provider teams working within flexible, integrated, interoperable, and digitally enabled care environments that strategically leverage digital tools, technologies and services to transform care delivery*	HIMMS (2020) [32]
*The field of knowledge and practice associated with the development and use of digital technologies to improve health*	World Health Organization (2021) [28]
